# Evaluating the Effectiveness of an Antimicrobial Stewardship Program on Reducing the Incidence Rate of Healthcare-Associated *Clostridium difficile* Infection: A Non-Randomized, Stepped Wedge, Single-Site, Observational Study

**DOI:** 10.1371/journal.pone.0157671

**Published:** 2016-06-16

**Authors:** Giulio DiDiodato, Leslie McArthur

**Affiliations:** Department of Pharmacy, Royal Victoria Regional Health Centre, Barrie, Ontario, Canada; University of Florida, UNITED STATES

## Abstract

**Background:**

The incidence rate of healthcare-associated *Clostridium difficile* infection (HA-CDI) is estimated at 1 in 100 patients. Antibiotic exposure is the most consistently reported risk factor for HA-CDI. Strategies to reduce the risk of HA-CDI have focused on reducing antibiotic utilization. Prospective audit and feedback is a commonly used antimicrobial stewardship intervention (ASi). The impact of this ASi on risk of HA-CDI is equivocal. This study examines the effectiveness of a prospective audit and feedback ASi on reducing the risk of HA-CDI.

**Methods:**

Single-site, 339 bed community-hospital in Barrie, Ontario, Canada. Primary outcome is HA-CDI incidence rate. Daily prospective and audit ASi is the exposure variable. ASi implemented across 6 wards in a non-randomized, stepped wedge design. Criteria for ASi; any intravenous antibiotic use for ≥ 48 hrs, any oral fluoroquinolone or oral second generation cephalosporin use for ≥ 48 hrs, or any antimicrobial use for ≥ 5 days. HA-CDI cases and model covariates were aggregated by ward, year and month starting September 2008 and ending February 2016. Multi-level mixed effect negative binomial regression analysis was used to model the primary outcome, with intercept and slope coefficients for ward-level random effects estimated. Other covariates tested for inclusion in the final model were derived from previously published risk factors. Deviance residuals were used to assess the model’s goodness-of-fit.

**Findings:**

The dataset included 486 observation periods, of which 350 were control periods and 136 were intervention periods. After accounting for all other model covariates, the estimated overall ASi incidence rate ratio (IRR) was 0.48 (95% 0.30, 0.79). The ASi effect was independent of antimicrobial utilization. The ASi did not seem to reduce the risk of *Clostridium difficile* infection on the surgery wards (IRR 0.87, 95% CI 0.45, 1.69) compared to the medicine wards (IRR 0.42, 95% CI 0.28, 0.63). The ward-level burden of *Clostridium difficile* as measured by the ward’s previous month’s total CDI cases (CDI Lag) and the ward’s current month’s community-associated CDI cases (CA-CDI) was significantly associated with an increased risk of HA-CDI, with the estimated CDI Lag IRR of 1.21 (95% 1.15, 1.28) and the estimated CA-CDI IRR of 1.10 (95% CI 1.01, 1.20). The ward-level random intercept and slope coefficients were not significant. The final model demonstrated good fit.

**Conclusions:**

In this study, a daily prospective audit and feedback ASi resulted in a significant reduction in the risk of HA-CDI on the medicine wards, however, this effect was independent of an overall reduction in antibiotic utilization. In addition, the ward-level burden of *Clostridium difficile* was shown to significantly increase the risk of HA-CDI, reinforcing the importance of the environment as a source of HA-CDI.

## Introduction

*Clostridium difficile* is a bacterium that can cause mild to severe and life-threatening diseases of the intestine [1]. *Clostridium difficile* infection is the most common healthcare-associated infection with an estimated incidence rate of up to 1 in 100 patients [2,3]. The attributable mortality rate of healthcare-associated *Clostridium difficile* infection (HA-CDI) is estimated to be 5.7% [4], and the attributable cost per incident case is estimated to be $10,809 (CDN), mostly due to increased length of hospital stay [2,3,5]. Public reporting of monthly HA-CDI incidence rates (cases per 1000 patient days) in acute care hospitals in Ontario has been mandatory since September 2008 [6].

The most consistently reported risk factors for HA-CDI include age over 65 years, prolonged hospitalization and recent antibiotic exposure [7–10]. Given that up to 50% of antibiotic use in hospitalized patients may be unwarranted [11], recent efforts to reduce the risk of HA-CDI have focused on curbing antibiotic utilization through the implementation of antimicrobial stewardship interventions (ASi) [12]. ASi refer to any activity that promotes the appropriate selection, dosing, route and duration of antimicrobial therapy with the intent of improving patient outcomes while reducing the risk of antimicrobial-associated infections such as HA-CDI [12].

Prospective audit and feedback is an ASi whereby dedicated healthcare providers review antimicrobial prescribing on a case-by-case basis in real time (audit) and then make recommendations to the attending physician regarding optimizing ongoing antimicrobial treatment (feedback) [13]. The attending physician then chooses to either accept or reject the recommendation(s). In a recent systematic review examining the effect of ASi utilizing a prospective audit and feedback strategy to reduce HA-CDI, five studies were identified that met the inclusion criteria and their pooled relative risk for HA-CDI was 0.49 (95% CI 0.24, 1.01) [14]. Three of the five studies were before-after studies that did not take temporal trends into account, and the other two studies were interrupted time series designs, each carried out in a single hospital, one in 3 intensive care units and the other in a geriatric ward enrolling only patients over 80 years old. There was significant heterogeneity among the results, with the 2 largest studies failing to demonstrate a reduction in the risk of HA-CDI. The post-intervention periods in these studies ranged from as low as 4 months to 21 months.

The exact mechanism by which ASi might reduce the incidence of HA-CDI is unclear, that is, whether the effect is at the patient-level and/or the ward- or hospital-level. The patient-level impact of an ASi might be to reduce individual antimicrobial exposure below a risk threshold such that HA-CDI is averted for that patient [15], whereas a ward- or hospital-level impact of an ASi might be to reduce environmental contamination with *Clostridium difficile* spores through an overall reduction in antimicrobial use resulting in fewer cases of HA-CDI [16].

A recent study has demonstrated the detrimental ecologic impact of antimicrobial use on HA-CDI incidence rates [10]. Brown *et al*. used a multilevel poisson regression model with random intercepts corresponding to wards to demonstrate that for every 10% increase in ward-level antimicrobial use, the incidence rate of HA-CDI increased by 34% (p<0.001) even after accounting for patient-level antimicrobial exposure [10]. The authors hypothesized that by exposing asymptomatically colonized patients with *Clostridium difficile* to antimicrobials there could be a resultant increase in antimicrobial-associated diarrhea leading to increased environmental contamination with *Clostridium difficile* spores. This increased environmental spore burden could then result in increased rates of subsequent colonization and infection of other hospitalized patients. This potential mechanism linking ward-level antimicrobial consumption to increasing rates of *Clostridium difficile* infection is supported by the observations that only 30% of all HA-CDI cases appear to be due to direct patient-patient transmission, whereas 45% of HA-CDI cases appear to be due to environment-patient transmission and the remainder have an unidentified primary source [17].

The effectiveness of ASi on reducing antimicrobial prescribing in individual patients has been recently reviewed [13]. In general, depending on the type of ASi implemented and the study design used to assess the ASi effect size, reductions in prescribing in individual patients have ranged from as low as 3.5% to as high as 42.3% [13]. However, no study to date has been able to demonstrate overall or hospital-wide reductions in antimicrobial use attributable to ASi [18–21]. The reason for this lack of ecological impact on antimicrobial use is that antimicrobial stewardship programs simply cannot review every patient prescribed antimicrobials given that on any given day in hospital it has been estimated that up to 50% of patients are prescribed at least one antimicrobial [22]. In addition, even if antimicrobial stewardship programs could review every antimicrobial prescription, the potential for intervention would be limited to 40% to 50% of prescriptions [11,23]. As a result, the current recommendation for antimicrobial stewardship programs is to focus ASi on high-risk antimicrobials that increase the risk for HA-CDI [23].

To summarize, the risk for HA-CDI is increased by both patient-level and hospital-level risk factors, especially antimicrobial use. Antimicrobial use is a modifiable patient-level risk factor that has been positively affected by ASi. The effectiveness of a prospective audit and feedback ASi to reduce HA-CDI is equivocal and may be dependent upon or interact with hospital-level antimicrobial use.

By focusing on high risk antimicrobial use in consecutive adult patients (≥18 years) admitted to any one of 6 wards (4 medical, 2 surgical) in a large community-based acute care hospital in Barrie, Ontario, does the implementation of an ASi utilizing a prospective audit and feedback strategy reduce the monthly ward incidence rate of HA-CDI independent of the total antimicrobial use on the hospital wards?

## Methods

### Setting

The study was conducted at the Royal Victoria Regional Health Centre (RVRHC), a 339-bed acute care hospital located in Barrie, Ontario. There were 6 wards that were exposed to the ASi: 4 general medicine wards (3GA, 3GC, 4GC, and 3SA) and 2 surgical wards (4SB and 4NC). These wards were chosen as they represent the wards with the highest antimicrobial use. While the medical wards generally admit similar types of patients, each ward has a tendency to serve slightly different medical populations. Respiratory illnesses (3GA), cancer and palliative care (3GC), cardiac disease (4GC) and geriatrics (3SA) are unique populations served by their respective wards. For the surgery service, the two surgical wards (4SB and 4NC) serve identical patients. The intensive care unit (ICU) served as a temporal control for any changes in hospital-related infection prevention and control practices that could impact the incidence rate of HA-CDI. The Royal Victoria Regional Health Centre Research Ethics Committee approved the study in January 2013. Waiver of informed consent was granted given that the antimicrobial stewardship program had already been approved for implementation as a clinical program and was identified as a required organizational practice by Accreditation Canada, the main accreditation agency for health care quality and patient safety in Canada [24].

### Study Design

**Fig 1:**
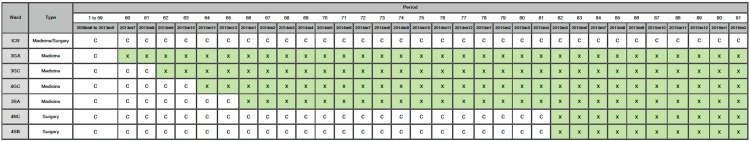
Stepped-wedge roll out of ASi by ward and month. C = control months and X = intervention months.

This was a non-randomized stepped wedge observational study [25]. Baseline aggregate data was collected for each ward on a monthly basis starting in September 2008 to June 2013. September 2008 was chosen as the start date to coincide with the first month of mandatory public reporting of HA-CDI by hospitals in Ontario. During this baseline period, no ward was exposed to the ASi. Starting July 2013, the ASi was rolled out to each medical ward in a non-randomized manner at 2 month intervals (Fig 1). By January 2014, ASi rollout to the medical wards was complete. The non-randomized order of the rollout of the ASi to the medical wards was done according to historical ward-level antimicrobial utilization, with the wards with the greatest antimicrobial use being the first to be receive the ASi. This was done for pragmatic reasons; to ensure maximal benefit to the most at-risk patients according to antimicrobial exposure, because the program needed to demonstrate its impact on patient safety to administrators in the shortest time period possible, admission to the medical wards is controlled by blinded administrators to the study, and hospitalists and internists care for these patients across all the medical wards so the value of randomizing the order of ward implementation in reducing selection bias is likely to have had minimal impact on the validity of the outcome. All medical wards continued to receive the ASi throughout the remainder of the study. The 2 surgical wards (along with the ICU) continued to serve as contemporaneous controls until May 2015, at which time the ASi was introduced to each surgical ward simultaneously. This delay was due to the manpower constraints of the stewardship program. The surgical wards continued to receive the ASi throughout the remainder of the study (end of February 2016).

### Intervention (ASi)

Prior to the ASi rollout, all the medical and surgical services’ healthcare providers (nurses and physicians) received an educational session detailing the goals of the antimicrobial stewardship program (reduce both high risk antimicrobial use and *Clostridium difficile* infection risk), in addition to a detailed explanation of the operational aspects of the prospective audit and feedback ASi.

All adult patients (≥18 years old) admitted to any of the study wards receiving the targeted antimicrobials were screened for review on a daily basis. Patients were identified using automated daily reports developed by the RVRHC decision support unit and clinical informatics service. The targeted antimicrobials included;

1. any antimicrobial that was administered intravenously for ≥ 48 hours, or
2. any fluoroquinolone (levofloxacin, moxifloxacin or ciprofloxacin) or second-generation cephalosporin (cefprozil or cefuroxime) that was administered orally for ≥ 48 hours, or
3. any antimicrobial that was administered for ≥ 5 days regardless of the route of administration.

The comprehensiveness of the daily automated reports to accurately identify ‘patients for review’ was assessed against a manual review of patient records by the antimicrobial stewardship team members on Medicine 3GA from April 1, 2013 to April 30, 2013, excluding Saturdays and Sundays. There was almost complete concordance between the two methods (Pearson’s correlation coefficient 0.94). Patients were reviewed on the day that they met the inclusion criteria except for Saturdays and Sundays; these patients were reviewed on the following weekdays if they still meet the ASi criteria since the ASi operated from Mondays to Fridays. Depending upon antimicrobial prescribing, patients may have been reviewed more than once during any hospital stay, however, only one ASi review per patient in any consecutive 12 month period was included in the study.

The appropriateness of antimicrobial prescribing was determined by members of the antimicrobial stewardship program (infectious diseases-trained pharmacist (Leslie McArthur) and infectious diseases physician (Giulio DiDiodato)) using evidence-based guidelines. The antimicrobial stewardship team members determined when an audit required review by both team members prior to making recommendations. ASi recommendations to the medical/surgical services followed those defined by Ashiuri *et al*. [26]. These included the following: No change to current antimicrobial(s) treatment, discontinue antimicrobial(s), intravenous to oral antimicrobial conversion (IV to PO), change duration of therapy or dosing, narrow or broaden spectrum of antimicrobial therapy or recommend an Infectious Diseases consultation. The ASi recommendations were not mutually exclusive, so more than one recommendation could be made for any ASi review. All recommendations were documented in the patient’s electronic medical record and communicated directly to the attending physician/surgeon by the antimicrobial stewardship team members. Acceptance or rejection of the ASi recommendation was documented in the patient’s electronic medical record. Acceptance (or rejection) was defined as occurring within 24 hours of the ASi recommendation(s).

### Outcome

The incidence rate of HA-CDI was the primary outcome. In accordance with the Ontario surveillance case definition, HA-CDI is defined as any positive result on a *Clostridium difficile* toxin or molecular assay of a stool specimen obtained from a patient with diarrhea (defined as 3 or more loose watery bowel movements in a 24-hour period) that develops ≥ 72 hours after admission to hospital (hospital-onset HA-CDI) or within 30 days after hospital discharge (community-onset HA-CDI) [27]. While this definition for HA-CDI did not change over the study period nor did the RVRHC policy mandating the submission of stool samples for testing in any patient who meets the diarrhea criteria, the microbiological test used to detect *Clostridium difficile* in stool samples did change during the course of this study. From September 2008 to June 2013, an enzyme-linked immunological assay was used (*C*. *DIFF QUIK CHEK COMPLETE*, Techlab), whereas from July 2013 to the end of the study, subsequent stool testing was done using a DNA-based assay (*GeneXpert*, Cepheid GeneXpert). The DNA-based assay is a much more sensitive assay than the enzyme-linked immunological assay and has been reported to result in an increase in the reported cases of CDI [28–30]. In this study, only cases acquired from the RVRHC were included in the HA-CDI counts. For example, if a patient was transferred from another acute care hospital to the RVRHC and developed CDI within 72 hours of admission to the RVRHC, then this patient would be considered to have HA-CDI not acquired from the RVRHC but rather from the sending facility and so would not be included in this study’s HA-CDI counts. All other cases of CDI that did not meet the criteria for either hospital-onset HA-CDI or community-onset HA-CDI were classified as community/unknown-associated CDI (CA-CDI). For any patient, only the first occurrence of HA-CDI in any consecutive 12 month period was included in the analysis to avoid misclassification between new/re-infection and relapsed infections [1].

### Data Sources

All the data were aggregated by ward, year and month. All the aggregated data were available from the RVRHC patient and administrative databases. All data was parsed into monthly segments to account for the monthly reporting of aggregated data. No direct or indirect patient health identifiers were included in the data or analyses. HA-CDI case ascertainment was done through the RVRHC patient and administrative database. Hospital-onset HA-CDI was defined as any positive microbiological stool sample for *Clostridium difficile* taken from an admitted patient at ≥ 72 hours after admission. The date that the stool sample was submitted to the microbiology laboratory was considered as the date of diagnosis. Over the course of the study period, stool sample testing for *Clostridium difficile* was done 7 days a week. Community-onset HA-CDI was defined as either:

1. any patient diagnosed with a positive microbiological stool sample for *Clostridium difficile* taken from an admitted patient at < 72 hours after admission who had been previously admitted to the RVRHC within the last 30 days, or
2. any patient with a discharge diagnosis of CDI (ICD-10 code A047) who had been previously admitted to the RVRHC within the last 30 days but did not have any positive microbiological stool sample for *Clostridium difficile* taken at ≥ 72 hours after admission or who did not have any in-hospital positive microbiological stool sample for *Clostridium difficile* at any time during their current admission. While all hospitals are required to prospectively perform HA-CDI surveillance and publicly report HA-CDI rates, the use of patient and administrative databases to ascertain the source of CDI has been validated in many studies [31–33]. In addition, the use of objective criteria for HA-CDI definitions through patient and administrative databases avoids any ‘gaming’ to the classification of the source of CDI that may occur by hospitals to improve their publicly reported HA-CDI rates.

### Statistical Analyses

The aggregated data were collected as cross-sectional, time-series data by ward, year and month. No continuous covariate distributions required transformations. HA-CDI cases were modeled as multilevel (monthly HA-CDI cases (level 1) and ward (level 2)) mixed effect count outcomes with poisson distribution (null model). Evidence for over dispersion was demonstrated by comparing the null model to the negative binomial distribution model using the likelihood ratio test (χ^2^ (df 1) = 12.43, p = 0.0002). The number of patient days at risk for HA-CDI (all patient hospital days ≥ 72 hours) represented the exposure time. Patient days after diagnosis of HA-CDI or CA-CDI were not included in the exposure time. Patient days due to re-admission(s) within 12 months of the primary admission were not included in the exposure time given recent hospital admission is a strong risk factor for HA-CDI. Patient days were parsed into monthly segments to account for the monthly reporting of aggregated data. Patient days were assigned to the wards where the length of stay was ≥ 72 hours. If patients were moved from one ward to another during the same admission, their patient days after transfer were only ascribed to their new ward after they had been admitted there for ≥ 72 hours. Robust standard errors were used to account for any residual potential clustering of unobserved confounders by ward.

The introduction of ASi on each ward was coded as a dichotomous exposure variable (control = 0, ASi = 1). The covariates considered for inclusion in the final multivariable multilevel negative binomial regression model had been previously shown to alter the risk for infection with *Clostridium difficile* and included; total antimicrobial use (days of therapy), total number of ASi consults (only the first prospective audit and recommendation for each patient in any consecutive 12 month period was included) and acceptance rate of ASi consults (% of total ASi consults), mean length of hospital stay (days), mean number of hospital admissions, proton pump inhibitor use (days of lansoprazole, pantoprazole, rabeprazole, omeprazole or esomeprazole), mean occupancy rate (% of total beds), total patient days spent in private rooms, total number of patient transfers, CDI environmental pressure defined as a combination of both the ward’s previous month’s total CDI cases and the ward’s current month’s CA-CDI cases (tested as separate covariates), service type (ICU, medical or surgical), and mean age (years). Evidence for effect modification was done by including interaction terms between the following: [ASi (yes/no) and total days of antimicrobial therapy], [ASi (yes/no) and total number of ASi consults], [ASi (yes/no) and acceptance rate of ASi consults], [total number of ASi consults and overall ASi consult acceptance rate], [total number of ASi consults and overall ASi consult acceptance rate and total days of antimicrobial therapy], [total days of antimicrobial therapy and total days of antimicrobial therapy in patients ≥ 65 years old], and [ASi (yes/no) and service type (medical or surgical)].

ASi and total days of antimicrobial use were forced into the final multivariable model because of their consistent relationship with HA-CDI [1], but all other covariates were assessed for inclusion in the final model. Univariate analysis using a p-value <0.2 as a cut-off significance value was used to identify covariates for possible inclusion in the final multivariable model. Forward selection process in the order determined from most significant to least significant p-values and size of univariate effect was used to determine the order of covariate inclusion in the final multivariable model. Final selection of covariates for inclusion was determined by using Akaike information criteria (AIC) and Bayesian information criteria (BIC) to compare goodness of fit of nested models.

In the multilevel model used in the analysis, the level 1 observations (monthly HA-CDI counts) were nested within the level 2 observations (individual wards). The random intercept for the wards will be reported along with the random slope for the unique effect, if any, of ASi (yes/no) at the ward level. The fixed coefficients of the covariates included in the final model were exponentiated and reported as incidence rate ratios (IRR) in the final model. An IRR of 1 indicates no relationship to the primary outcome, whereas an IRR > 1 indicates that a unit change in the covariate increases the incidence rate of HA-CDI and an IRR < 1 indicates that a unit change in the covariate decreases the incidence rate of HA-CDI. Analysis was done using a generalized linear model with a binomial-log family-link function using STATA 14.1.

## Results

### Setting

**Fig 2:**
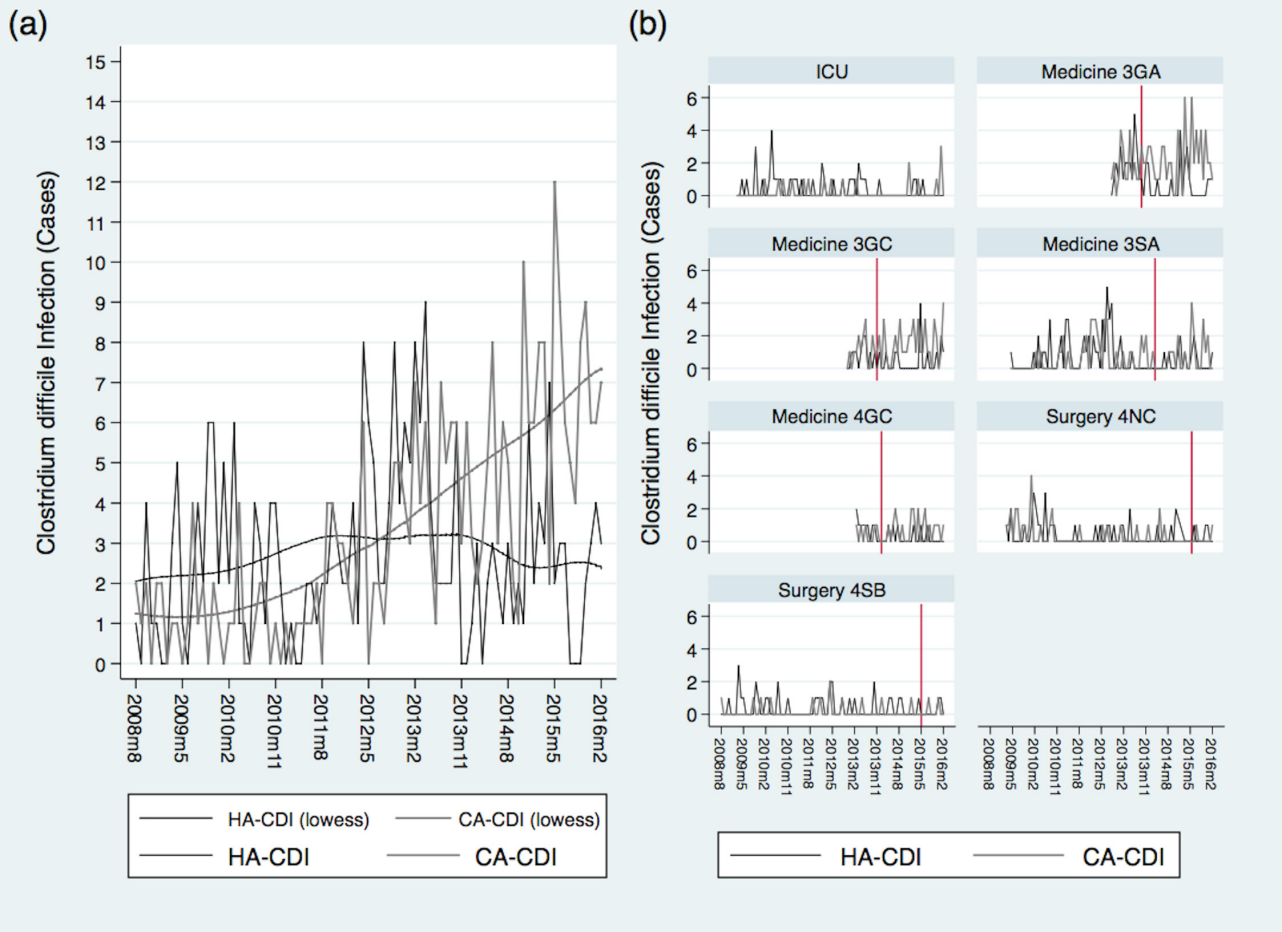
**Temporal trends of HA-CDI and CA-CDI by (a) total, and (b) ward.** The smooth trend line in (a) was calculated from the time series using a locally weighted smoothing function (lowess). The vertical reference lines in (b) represent the start date for ASi for that ward.

Over the course of the study period, the hospital underwent an expansion that explains the imbalance in the number of observations for each ward. The temporal trends of HA-CDI and CA-CDI cases fluctuated significantly by ward and time period (Fig 2).

The total number of patients admitted over the study period was 58 261, contributing 201 076 at-risk patient days and consuming 118 494 days of antimicrobial therapy. There were a total of 249 HA-CDI cases over the study period, with 38 cases in the ICU, 142 cases on the medicine service and 69 cases in the surgery service. The monthly covariate values varied considerably between the three services; ICU, medicine and surgery (Table 1).

**Table 1:** Comparison of the monthly variable averages between the ICU, medical and surgery services.

Variables	ICU	Medicine	Surgery
	Monthly Averages (standard deviation)		
**HA-CDI (cases)**	0.42(0.70)	0.66(0.99)	0.38(0.66)
**CA-CDI (cases)**	0.19(0.49)	1.09(1.21)	0.24(0.57)
**Exposure (Patient days at risk for CDI)**	222(86.1)	489(194.7)	420(132.7)
**Antibiotic treatment (days of therapy)**	152(41.1)	240(93.8)	295(68.3)
**Proton pump inhibitor (days of therapy)**	88(34.8)	243(94.7)	314(100.2)
**Length of stay (days)**	4.63(1.03)	5.94(3.61)	2.85(0.50)
**Age (years)**	61(2.5)	71(6.7)	61(2.5)
**Ward occupancy rate (%)**	71(10.1)	101(3.6)	80.6(6.6)
**Private room (days)**	34(27.6)	229(146.9)	77.5(28.7)
**Patient in-hospital transfers (number)**	487(420)	715(422)	555(292)
**Patient admissions (number)**	56(10.8)	90(35.4)	188(35.7)

### Antimicrobial Stewardship Activity

**Fig 3:**
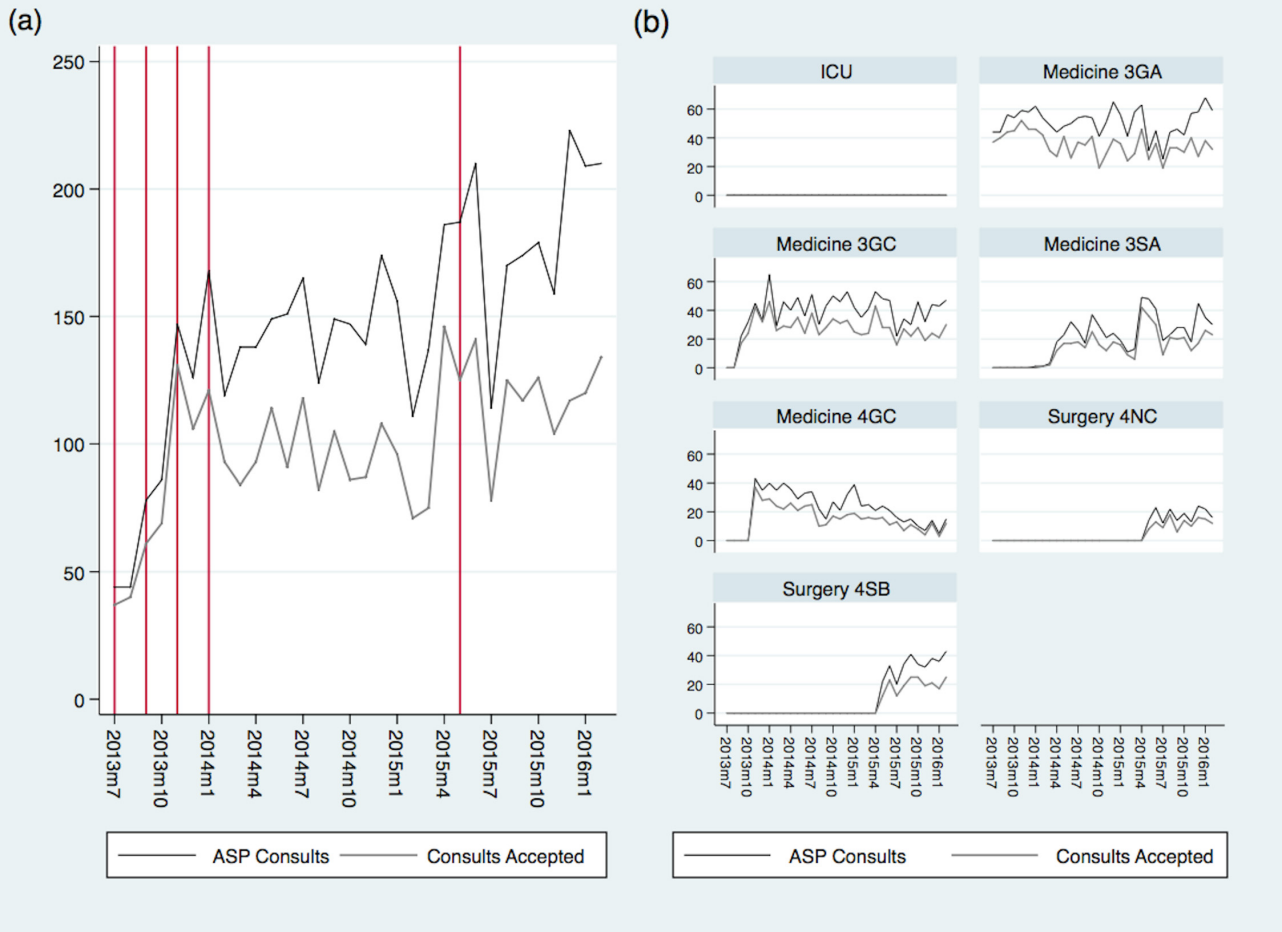
**Temporal trends of ASi consults and number accepted by (a) total, and (b) ward.** The vertical reference lines in (a) represent the different start dates for ASi.

The overall number of ASi consults and the number of ASi consults accepted varied significantly over time and by ward (Fig 3).

The majority of ASi consults resulted in the discontinuation of antimicrobials, especially of the quinolone and cephalosporin classes (Table 2). The total number of unique ASi consults was 3 750 for the medicine service and 447 for the surgery service. The overall ASi recommendation acceptance rate was 67.4%, with 68.1% for the medicine service and 60.6% for the surgery service.

**Table 2:** Types of ASi recommendations and their acceptance, by service.

ASi Recommendation^a^	Medicine	Surgery
	(ASi Recommendation/Number Accepted)	
**Discontinue Antimicrobial(s)**	1537/1225	166/112
**Quinolone**	587	56
**Cephalosporin**^b^	560	41
**IV to PO**	697/584	75/57
**Narrow spectrum**	644/559	51/42
**Broaden spectrum**	143/129	43/39
**Optimize Duration**	574/471	36/22
**Dose Adjustment**	160/146	34/31
**No change**	661/621	90/82
**Infectious Diseases consult**	177/144	13/10

^a^ Patients may have had more than one ASi recommendation at the time of the ASi consult.

^b^ Includes second and third generation (ceftriaxone, cefotaxime and ceftazidime) cephalosporins.

### Multilevel Mixed-Effects Modeling of HA-CDI

#### Covariate distributions in control and ASi periods

The baseline risk of infection in the control period varied considerably between the different medical wards ranging between 0.77 to 1.85 cases per month, compared to the relatively low and consistent risk seen in both surgery wards (Table 3). The lower risk of HA-CDI in the surgery wards was partly attributable to the short length of stay whose average was below the threshold needed to meet the diagnostic criteria for infection. The number of observation periods in the control phase ranged from 11 (Medicine 4GC) to 81 (Surgery 4SB), while the observation periods in the ASi phase ranged from 10 (Surgery 4SB and Surgery 4NC) to 32 (Medicine 3GA). The reason for these uneven observation periods was due to a hospital expansion that occurred during the study period.

**Table 3:** Variable distributions (Mean (standard deviation)) in Control and ASi Periods, by ward.

Variables	Medicine 3GA		Medicine 3GC		Medicine 4GC		Medicine 3SA		Surgery 4NC		Surgery 4SB	
	Control	ASi	Control	ASi	Control	ASi	Control	ASi	Control	ASi	Control	ASi
**HA-CDI**	1.85 (1.28)	0.62 (1.0)	0.77 (0.72)	0.43 (0.86)	0.82 (0.60)	0.21 (0.42)	0.78 (1.17)	0.42 (0.64)	0.41 (0.71)	0.30 (0.48)	0.40 (0.66)	0.20 (0.42)
**CA-CDI**	1.69 (1.25)	2.25 (1.50)	1.07 (0.95)	1.50 (1.11)	0.45 (0.52)	0.71 (0.76)	0.62 (0.94)	0.69 (1.09)	0.36 (0.72)	0.20 (0.42)	0.15 (0.39)	0.20 (0.42)
**Exposure**	437 (109)	563 (77.3)	449 (120)	592 (127)	594 (105)	729 (128)	299 (137)	485 (191)	398 (147)	506 (89.1)	420 (123)	497 (50.4)
**Antibiotic treatment**	370 (90.4)	320 (40.9)	240 (59.9)	274 (60.8)	240 (56.3)	214 (54.0)	195 (98.9)	176 (81.1)	293 (57.1)	211 (51.2)	311 (73.7)	263 (46.8)
**Proton pump inhibitor**	230 (49.8)	263 (55.3)	217 (60.4)	268 (52.1)	302 (36.4)	342 (58.4)	169 (83.5)	257 (133)	281 (74.7)	409 (67.9)	351 (100)	182 (78.5)
**Length of stay**	5.08 (0.49)	4.26 (0.69)	5.61 (0.77)	4.62 (1.00)	4.68 (0.46)	4.54 (0.76)	8.66 (5.59)	5.58 (2.0)	2.99 (0.52)	2.00 (0.00)	2.9 (0.31)	2.00 (0.00)
**Age**	65.6 (2.1)	65.4 (2.8)	65.3 (1.8)	65.9 (1.7)	68.4 (2.5)	69.8 (1.3)	78.6 (3.8)	78.1 (5.3)	60.8 (2.9)	65.8 (1.5)	62.0 (1.5)	59.4 (1.9)
**Ward occupancy rate**	98.3 (2.0)	102 (3.9)	95.7 (3.7)	101 (3.3)	98.0 (2.2)	103 (2.0)	101 (1.6)	103 (5.0)	77.4 (4.4)	90.6 (10.7)	81.2 (4.7)	92.3 (5.4)
**Private room**	263 (65.9)	347 (41.3)	257 (65.5)	362 (66.2)	309 (64.6)	384 (50.4)	49.8 (28.7)	131 (83.7)	81.3 (26.8)	104.6(25.2)	65.4 (23.1)	118 (25.7)
**Patient in-hospital transfers**	884 (153)	858 (126)	670 (163)	741 (152)	1350 (185)	1337 (176)	254 (235)	622 (281)	496 (312)	895 (99.1)	539 (262)	795 (114)
**Patient admissions**	98.4 (18.9)	104 (12.8)	86.1 (18.9)	100 (15.4)	137 (15.8)	131 (17.7)	57.6 (28.7)	74.4 (35.9)	174 (38.5)	206 (16.6)	197 (31.3)	208 (16.9)
**ASi consults**	0	51.1 (9.5)	0	41.1 (9.8)	0	24.7 (10.8)	0	24.6 (13.2)	0	17.9 (4.6)	0	33.3 (7.4)
**ASi consults accepted**	0	35.1 (8.2)	0	28.3 (7.3)	0	16.7 (8.0)	0	16.9 (9.9)	0	12.1 (3.8)	0	19.8 (5.0)
**ObservationPeriods (Months)**	13	32	13	30	11	28	63	26	78	10	81	10

### Univariate Analysis

Univariate analyses demonstrated that the average age and average ward occupancy rate did not seem to be significantly related to HA-CDI, but all the other covariates were tested for inclusion in the final model (Table 4). The order of covariate entry into the final model was based on the P-value and the effect size. For example, LOS (length of stay) was tested before CA-CDI even though the CA-CDI effect size was larger but its P-value was less significant than that of LOS. The baseline incidence rate for HA-CDI was estimated to be 1.25 per 1 000 patient days (95% CI, 0.86–1.82).

**Table 4:** Univariate analyses of HA-CDI dependence on covariates.

Covariates	Coefficient	Robust SE	P>|z|	95% confidence interval	
**Null**^1^	-6.6819	0.1900	0.0000	-7.0543	-6.3096
**ASi**^2^	Forced into final model				
**Antibiotic**^3^	Forced into final model				
**PPI**^4^	-0.0021	0.0014	0.1260	-0.0048	0.0006
**LOS**^5^	0.0319	0.0171	0.0630	-0.0017	0.0655
**Age**^6^	-0.0068	0.0258	0.7920	-0.0574	0.0438
**Occupancy**^7^	-0.0032	0.0083	0.7010	-0.0194	0.0130
**Private Rm**^8^	-0.0056	0.0011	0.0000	-0.0077	-0.0034
**Transfers**^9^	-0.0013	0.0002	0.0000	-0.0018	-0.0009
**Admissions**^10^	-0.0067	0.0016	0.0000	-0.0098	-0.0035
**ASi Consults**^11^	-0.0200	0.0035	0.0000	-0.0268	-0.0132
**ASi Accept**^12^	-0.0156	0.0021	0.0000	-0.0197	-0.0114
**CDI Lag**^13^	0.2274	0.0424	0.0000	0.1443	0.3105
**Service**^14^	-0.3752	0.0660	0.0000	-0.5046	-0.2458
**CA-CDI**^15^	0.0712	0.0454	0.1170	-0.0178	0.1601

^1^Null model includes only HA-CDI intercept

^2^ASi is a dichotomous covariate, with control period = 0 and ASi period = 1

^3^Antibiotic refers to total days of antimicrobial therapy

^4^PPI refers to total days of proton pump inhibitor therapy

^5^LOS refers to average length of stay in days

^6^Age refers to average age in years

^7^Occupancy refers to average ward occupancy rate

^8^Private Rm refers to total days patients were admitted to private rooms

^9^Transfers refers to total number of patient transfers from their rooms. Transfers could be due to need for diagnostic imaging, isolation, surgery, etc…

^10^Admissions refers to total number of patients admitted to ward

^11^ASi Consults refers to total number of unique ASi consults

^12^ASi Accept refers to rate (percentage) of ASi recommendations accepted

^13^CDI Lag refers to each ward’s previous month’s total CDI count (HA-CDI plus CA-CDI)

^14^Service refers to three services categorized as: ICU (= 0), medicine (= 1) and surgery (= 2)

^15^CA-CDI refers to each ward’s current month’s CA-CDI count

### Multivariate Analysis

The final model included 9 covariates (Table 5); PPI, ASi consults, ASi accept were not included in the final model (data not included in Table 5). The random intercept (ward-level variation of HA-CDI rate) and random slope (ward-level variation of ASi effect) coefficients were not significant. All interaction terms that were tested for inclusion did not improve the final model and were discarded (data not included in Table 5). The AIC and BIC values for the null model were 972.1 and 984.7, respectively, while the final model’s AIC and BIC values were 891.8 and 916.9, respectively.

**Table 5:** Multivariate analysis of HA-CDI dependence on covariates identified from univariate analyses.

Covariates	IRR^a^	Robust SE	P>|z|	95% confidence interval	
**Null**	0.0028	0.0004	0.0000	0.0021	0.0037
**Antibiotic**	1.0025	0.0011	0.0300	1.0002	1.0047
**LOS**	0.9701	0.0056	0.0000	0.9591	0.9811
**Private Rm**	0.9991	0.0008	0.2770	0.9976	1.0007
**Transfers**	0.9994	0.0003	0.0230	0.9989	0.9999
**Admissions**	0.9939	0.0019	0.0020	0.9902	0.9977
**CDI Lag**	1.2136	0.0348	0.0000	1.1473	1.2838
**Service-Medicine**	1.0027	0.1484	0.9850	0.7503	1.3401
**Service-Surgery**	0.6785	0.1264	0.0370	0.4710	0.9775
**CA-CDI**	1.1045	0.0476	0.0210	1.0150	1.2019
**ASi Effect**					
**Overall**	0.4836	0.1201	0.0030	0.2972	0.7869
**Medicine**	0.4243	0.0857	0.0000	0.2856	0.6305
**Surgery**	0.8701	0.2946	0.6810	0.4480	1.6896

^a^IRR refers to incidence rate reduction

#### Goodness-of-fit

Deviance residuals for the final model were calculated and demonstrated good fit.

## Discussion

In this 91-month non-randomized, stepped wedge observational study, we demonstrated an overall reduction in risk of HA-CDI of 51.6% (95% CI, 21.3%-70.3%) in those wards exposed to our ASi. Ward-level antimicrobial use is a significant risk factor for HA-CDI. According to the model, for every 50 days of antimicrobial therapy, the risk for HA-CDI increases by 13.3% (95% CI, 1%-26%). The average monthly days of antimicrobial therapy in our study was 244 (sd 92). The ward-level effect of antimicrobial utilization on rates of HA-CDI predicted by our model is significantly less that that predicted by Brown *et al*. who estimated a 34% (95% CI, 16%-57%) increase in HA-CDI infection risk for every 10% increase in ward-level antimicrobial utilization [10]. However, that study did not explore the potential impact of CDI environmental pressure or antimicrobial stewardship interventions on the risk for HA-CDI, thus likely overestimating the attributable risk due to ward-level antimicrobial exposure.

To our knowledge, this is the first study to estimate the impact of CDI environmental pressure on subsequent risk of HA-CDI after accounting for both antimicrobial use and antimicrobial stewardship activities [34–38]. In this study, CDI environmental pressure was defined as the ward-level prevalence of total CDI cases (both HA-CDI and CA-CDI) and CA-CDI cases in the preceding month and current month, respectively. One additional CDI case in either the previous month’s CDI cases (HA-CDI and CA-CDI) or the current month’s CA-CDI cases increased the risk for HA-CDI by 21.9% (95% CI, 14.7%-28.4%) and 10.4% (95% CI 1.5%-20.2%), respectively. This is consistent with our emerging understanding of the epidemiology of HA-CDI that suggests the most important reservoir for *Clostridium difficile* is the hospital environment which becomes contaminated by highly resistant *Clostridium difficile* spores via symptomatic and asymptomatic fecal shedders [7,16,17,39,40]. Hospitalized patients subsequently ingest these spores, and given other risk factors such as antimicrobial use or proton pump inhibitor treatment, develop *Clostridium difficile* infection.

There are several risk reduction strategies for *Clostridium difficile* infection, including antimicrobial stewardship activities. While many studies have demonstrated the positive impact of ASi on reducing targeted antimicrobials, the overall effect of ASi on the risk of HA-CDI has not been unequivocally demonstrated for ASi that employ a prospective audit and feedback strategy [14,41,42]. In addition, others have modeled that a small reduction in high-risk antimicrobial (for causing HA-CDI) use by ASi could have a significant impact on reducing the risk of CDI [23]. Our model estimated an overall reduction in risk of HA-CDI of 51.6% (95% CI, 21.3%-70.3%) in those wards exposed to our ASi. This protective ASi effect was independent of the amount of ward-level antimicrobial use, the number of ASi prospective audits conducted and the number of ASi recommendations accepted by the attending physicians and surgeons. This observation can be explained in the following way; in the process of conducting as many prospective ASi audits as possible, the antimicrobial stewardship program only rarely intervenes in a high-risk patient and prevents that patient from developing CDI. By preventing these sporadic cases, the overall effect on the incidence of HA-CDI is significant despite the absence of any significant relationship between the ASi effect and the activity level of the antimicrobial stewardship program or the amount of overall antimicrobial utilization. This hypothesis is consistent with our emerging understanding of the epidemiology of HA-CDI [17,23,40]. In addition, this hypothesis is also supported by the findings of a recent study in a long-term acute care hospital that demonstrated very few ASi prospective audits (885 audits conducted in a 212 bed facility over a period of 3 years) associated with low ASi recommendation acceptance rates ranging from 12.5% to 60% resulted in a reduction in the incidence rate of HA-CDI by 43% (95% CI 8%-65%) [32]. The protective effect of ASi was only significant in medicine patients with an estimated reduction in CDI infection risk of 57.5% (95% CI, 37.0%-71.4%). While there were 20 months of ASi exposure data for the surgery service which should have been sufficient to detect a significant ASi effect, the lack of ASi effect in our surgery patients could be due to the fact that they seem to be at an estimated lower risk for HA-CDI than our medicine patients by 32.3% (95% CI 27.1%-37.2%). Antimicrobial stewardship programs could use this information to prioritize their activities especially if stewardship resources are limited.

Both private room utilization and increased numbers of admissions were protective against infection, while decreasing the number of transfers and the length of hospital stay seemed to put patients at risk for HA-CDI. This last finding is paradoxical, and could be attributable to unobserved competing events. For example, patients who are more severely ill may have both fewer in-hospital transfers and shorter lengths of stay due to death but may be at higher risk for HA-CDI.

While this was a single site study, the goal of the study was to determine the effectiveness of a local antimicrobial stewardship program in reducing the risk of HA-CDI in our patients. For our hospital, we have a duty to our administrators, physicians, and patients to demonstrate that our activities and program costs are effective and efficient and improve patient safety and quality of care. This study has served that purpose well. This study could be used as a model by other antimicrobial stewardship programs that wish to conduct their own methodologically robust studies and evaluations to help support their own programs. The study design is robust despite being an observational study; it had a contemporaneous control ward (ICU) throughout the study period to account for any system-level changes that did occur (change in *Clostridium difficile* diagnostic test) or may have occurred but were unobserved, all the data is objective and readily available from our electronic database, and the periods of observation for both the control and ASi periods were significant making any findings resistant to spurious associations. In addition, while the increase in CA-CDI counts after the change from an enzyme-linked immunoassay to the more sensitive DNA-based assay system was expected [28–30], the HA-CDI counts did not experience a similar increase, thus reinforcing the likelihood that the reduction in the risk of HA-CDI after introduction of the ASi was substantial and authentic. However, as with any study where treatment allocation is neither properly randomized nor blinded, and outcome assessment is not blinded, there exists significant opportunity for bias in the estimated treatment effect.

## Conclusions

Our 91-month observational study of the incidence of HA-CDI across 6 wards in our large community-based hospital revealed the positive impact that our antimicrobial stewardship program has on patient safety and quality of care, an effect that seems to be independent of total antimicrobial utilization, or the activity level or acceptance rate of our antimicrobial stewardship recommendations. The current emphasis by governmental agencies and hospital administrators on increasing compliance with alcohol-based hand rubs to reduce the risk of HA-CDI is misplaced given the lack of evidence supporting this practice [10,43–46]. Instead, the importance of environmental *Clostridium difficile* pressure in increasing the risk of HA-CDI as estimated by our model should be used to promote a greater emphasis on environmental cleaning and early isolation of patients with diarrhea, along with universal glove and hand washing with soap and water to protect against contamination by *Clostridium difficile* spores. Antimicrobial stewardship programs seem to positively impact important patients outcomes, but the mechanism by which the ASi achieve these effects remains largely unknown. This study suggests that sporadic but important ASi on the ‘right patient’ is the route by which antimicrobial stewardship programs improve patient safety and quality of care. Until antimicrobial stewardship programs can better predict who the ‘right patient’ is, it looks as though for the near future that our efforts as stewards will continue to involve the review of large numbers of patients with the hope that the ‘right patient’ is included in our audits.

## Supporting Information

S1 Table Data File. Data file for all variables by ward, month and year.
(XLSX)
